# Nanoparticles targeting monocytes and macrophages as diagnostic and therapeutic tools for autoimmune diseases

**DOI:** 10.1016/j.heliyon.2023.e19861

**Published:** 2023-09-07

**Authors:** Karen Álvarez, Mauricio Rojas

**Affiliations:** a Grupo de Inmunología Celular e Inmunogenética, Sede de Investigación Universitaria (SIU), Universidad de Antioquia (UDEA), Colombia; b Unidad de Citometría de Flujo, Sede de Investigación Universitaria (SIU), Universidad de Antioquia (UDEA), Colombia

**Keywords:** Autoimmune diseases, Monocytes, Macrophages, Nanoparticles

## Abstract

Autoimmune diseases are chronic conditions that result from an inadequate immune response to self-antigens and affect many people worldwide. Their signs, symptoms, and clinical severity change throughout the course of the disease, therefore the diagnosis and treatment of autoimmune diseases are major challenges. Current diagnostic tools are often invasive and tend to identify the issue at advanced stages. Moreover, the available treatments for autoimmune diseases do not typically lead to complete remission and are associated with numerous side effects upon long-term usage. A promising strategy is the use of nanoparticles that can be used as contrast agents in diagnostic imaging techniques to detect specific cells present at the inflammatory infiltrates in tissues that are not easily accessible by biopsy. In addition, NPs can be designed to deliver drugs to a cell population or tissue. Considering the significant role played by monocytes in the development of chronic inflammatory conditions and their emergence as a target for extracorporeal monitoring and precise interventions, this review focuses on recent advancements in nanoparticle-based strategies for diagnosing and treating autoimmune diseases, with a particular emphasis on targeting monocyte populations.

## Graphical abstract

Monocytes and macrophages are potential targets for nanoparticles for the diagnosis and treatment of autoimmune diseases. Different types of NPs with superparamagnetic properties can be used as contrast agents for early diagnostic imaging of different diseases. For this purpose, NPs are administered intravenously in different animal models or even in humans with autoimmune diseases (1) to be taken up by monocytes (2) that subsequently migrate to sites of inflammation in response to chemotactic signals (3). Thus, monocytes that have captured NPs with superparamagnetic properties can be tracked *in vivo* by MRI (4) to visualize their early accumulation at sites of inflammation such as the CNS (multiple sclerosis), pancreas (diabetes mellitus), or inflamed synovial tissue (rheumatoid arthritis). Furthermore, as NPs can be loaded with drugs, peptides, and oligonucleotides, they can be used to specifically modulate the inflammatory function of monocytes and macrophages without compromising the function of other cells. NPs loaded with therapeutic agents increase the local concentration of the drug and reduce its side effects by lowering its systemic level (5). Created with BioRender.com.

**Figure:**
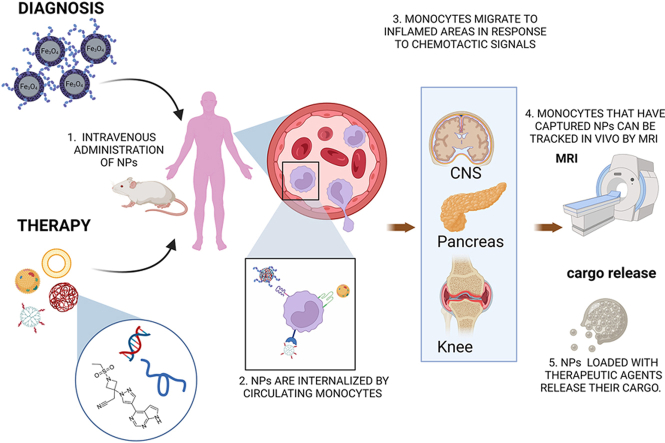

## Abbreviations

CNS: central nervous system
CSF1R: colony stimulating factor 1 receptor
EAE: experimental autoimmune encephalomyelitis
FRs: folate receptors
HEK: human embryonic kidney
ICAM-1: intercellular adhesion molecule
IL: interleukin
LPS: lipopolysaccharide
Mac-1: macrophage-1 antigen
MCP-1: monocyte chemoattractant protein-1
MCSFR: macrophage colony-stimulating factor receptor
mAb: monoclonal antibody
MRI: magnetic resonance imaging
mRNA: messenger RNA
MS: multiple sclerosis
NF-κB: nuclear factor kappa B
NOD: non-obese diabetic
NPs: nanoparticles
PLA: poly (D-1-lactic acid)
PLGA: poly (D-L-lactic co-glycolic acid)
RA: rheumatoid arthritis
ROS: reactive oxygen species
siRNA: small interfering ribonucleic acid
SLE: systemic lupus erythematosus
SPION: standard Superparamagnetic iron oxide nanoparticles
SS: Sjögren's syndrome
TCR: T-cell receptor
TD1: type 1 diabetes
TGF-β: transforming growth factor-β1
TNF-α: tumor necrosis factor-alpha
USPIO: ultrasmall Superparamagnetic iron oxide nanoparticles
VSPIO: very small Superparamagnetic iron oxide nanoparticles

## 1 Introduction

Autoimmune diseases, such as rheumatoid arthritis (RA), systemic lupus erythematosus (SLE), Sjögren's syndrome (SS), and multiple sclerosis (MS), are chronic conditions that result from an inadequate immune response to self-antigens [1]. Diagnosis of these illnesses is challenging, as their signs and symptoms can vary widely, and their severity can change over time. It often requires a combination of several diagnostic approaches and, in some cases, very invasive procedures, such as biopsying and coronary angiography [2,3]. Regarding the treatment of autoimmune diseases, it is important to mention the current availability of various immunosuppressive therapies that help improve patients' quality of life. However, the long-term use of these therapeutic agents may cause side effects leading to progressive health deterioration. For example, systemic glucocorticoids and antimetabolites may cause renal, neurological, hematological, and immunological toxicity [[4], [5], [6]]. In many cases, reduction of appropriate drug levels or combined therapies is required, even if the desired effects are not achieved. Therefore, the main challenges in this field are developing more sensitive diagnostic tools to facilitate early diagnosis of autoimmune diseases and more selective, timely, and effective treatments for the affected patients.

Nanoparticles (NPs) have become a promising tool for improving diagnostic and therapeutic approaches for autoimmune conditions. NPs are ultrafine particles whose three dimensions are in the nanoscale regime (generally between 1 and 100 nm) and have a wide variety of physicochemical characteristics in terms of size, shape, surface charge, hydrophobicity, and chemical composition [7]. NPs have been widely used in biomedicine as contrast agents for diagnostic imaging methods, as platforms for drug loading and delivery, and as tools for diagnosing different conditions. They also make it possible to monitor molecular and cellular changes associated with a disease state [8,9].

**Fig. 1:**
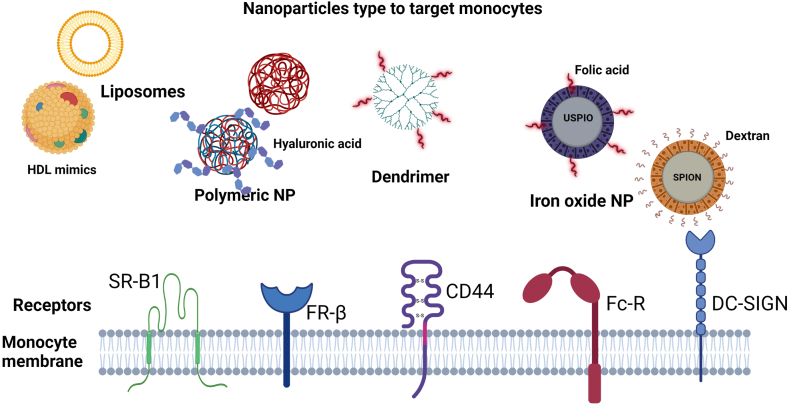
**Nanoparticles type to target monocytes and their receptors.** According to their chemical properties, NPs can be classified into different categories; liposomes, polymeric NPs, and iron oxide NPs are the most widely used in the diagnosis and treatment of autoimmune diseases. NPs can be functionalized by coating with different ligands (folic acid, dextran, antibodies, and other ligands) that mediate their recognition and internalization by monocytes and macrophages through different surface receptors, including scavenger (*e.g*., scavenger receptor class B type I), DC-SIGN, CD44, Fc and folate receptors. Created with BioRender.com.

There are different groups of NPs, including polymeric, inorganic, lipid-, and protein/peptide-based [10]. The most widely used in biomedicine comprise the following (Fig. 1): (i) Lipid-based NPs (liposomes, micelles, wrapsomes), which are spherical vesicles with one or more phospholipid bilayers that self-assemble in aqueous systems. Since the lipids in these NPs are like those found in the cell membrane, they can be more easily internalized by cells. In comparison to conventional drug delivery methods, these systems exhibit lower toxicity and allow for the administration of higher drug doses [10]. Additionally, their amphipathic nature allows them to interact with and transport hydrophilic compounds within the NPs and carry hydrophobic compounds embedded in the lipid bilayer [6,7]; (ii) NPs made from synthetic polymers such as poly(D-1-lactic acid) (PLA) and poly(D-L-lactic co-glycolic acid) (PLGA) or natural polymers such as chitosan and collagen. Given the wide diversity of polymer combinations, the variations in the composition and properties of polymeric NPs are unlimited, drugs can be encapsulated within the polymer or displayed on its surface [10], and (iii) iron oxide NPs with an iron core in different oxidation states such as magnetite or maghemite; they also have a hydrophilic layer of dextran or other biocompatible components that increase their stability and functionalization and reduce their cytotoxicity. Their low toxicity, biodegradability, and ease of manufacture make them an excellent choice for drug delivery and as contrast agents for magnetic resonance imaging (MRI). These NPs are classified into different categories according to their hydrodynamic diameter, as follows: Standard (SPION; (50–180 nm), ultrasmall (USPIO; 10–50 nm), and very small (VSPIO: <10 nm) superparamagnetic iron oxide NPs [7,11].

The physicochemical characteristics of NPs are determinants of their function and interaction with different cell types. For example, the size of NPs determines their biodistribution to different tissues. NPs smaller than 20 nm in size can pass through the walls of blood vessels, the blood-brain barrier, and the epithelium of the stomach and can therefore be used as contrast agents to examine different organs by imaging. In contrast, larger NPs can be efficiently taken up by phagocytic cells. Beduneau et al. reported that human monocytes and monocyte-derived macrophages captured 394-nm-diameter superparamagnetic iron oxide NPs more efficiently than smaller 62-nm-diameter NPs [12]. In addition, charge also plays an essential role in NPs uptake. Juliano et al. demonstrated that unilamellar liposomes were cleared from mouse plasma faster than multilamellar ones. They also found that positively charged unilamellar liposomes remained in circulation longer than negatively charged ones [13]. Similarly, rabbit monocytes [14] and mononuclear cells of the mouse phagocytic system [15] cleared negatively charged liposomes more efficiently than neutral ones. These results suggest that NPs with a negatively charged surface are taken up more efficiently than neutral NPs of the same size.

Recently, numerous reports have been published on the application of NPs in various fields such as pharmacology, photodynamic therapy, biosensing, and biochips [16]. NPs have been used for the detection of diverse types of cancers, tumor markers, pathogens [16,17], and autoimmune diseases [18,19]. Encapsulation of therapeutic agents into NPs prevents them from interacting with plasma proteins, such as complement factors and immunoglobulins, and protects them against enzymatic degradation. In addition, it allows the therapeutic agent to be targeted to a specific site, thus increasing its local concentration, and reducing the side effects caused by a high circulating level or interactions with cells or tissues not involved in the event [6]. The chemical nature of the surface of NPs determines their compatibility with immune system cells; however, they can be coated with different types of molecules to target a specific organ or cell population. In addition, NPs are a valuable tool for the intravenous administration of poorly soluble drugs that pose a significant challenge to the pharmaceutical industry as their poor aqueous solubility leads to low bioavailability and absorption. Water-insoluble drugs can be dispersed in aqueous solutions by encapsulation in NPs composed of long alternating sequences of two or more amphipathic monomers. These monomers assemble in water to form a structure with a hydrophilic corona, allowing it to interact with aqueous environments and a hydrophobic core where water-insoluble drugs can be encapsulated.

Circulating monocytes are a subpopulation of bone marrow-derived leukocytes capable of differentiating into macrophages and dendritic cells. They constitute an essential link between innate and adaptive immunity and accomplish important immunological functions such as phagocytosis, antigenic presentation, production of soluble mediators, initiation and resolution of inflammation, and recruitment of other immune system cells. Several studies of autoimmunity in animal models and patients with autoimmune diseases have described alterations in the phenotype, function, and activation of monocytes and macrophages [20,21]. These findings have highlighted the importance of monocytes/macrophages in the immunopathogenesis of autoimmune diseases and postulated them as potential targets for improving the diagnosis of autoimmune disorders and the treatment of the affected patients. As monocytes/macrophages have endocytic and phagocytic properties, they can be targeted by ingested NPs. However, since they express a wide repertoire of receptors (scavenger receptors, Fc receptors, lectin-like receptors, integrins) and a variety of lipids and proteins, NPs could be coated with specific antibodies or other ligands to facilitate their interaction with monocytes [22].

This review describes experimental evidence on the specific uptake of NPs by monocytes/macrophages, the potential utility of NPs for *in vivo* monitoring of monocytes/macrophages by MRI, and the *in vitro* and *in vivo* modulation of the inflammatory function of monocytes/macrophages by NPs carrying different drugs, peptides, and oligonucleotides (See Table 1).

**Table 1:** Applications of nanoparticles in the diagnosis and therapy of autoimmune diseases.

		Disease	Effects	Reference
**Diagnosis**	Dextran-coated magnetofluorescent	Type 1 diabetes (T1D)	Accumulation of nanoparticles uptaken by invading macrophages in pancreatic islets in mouse models of T1D detected by MRI. Accumulation was correlated with the aggressivity of the islet lesion.	[23]
	Monocrystalline superparamagnetic iron oxide NPs•			[24]
	Ferumoxtran-10 (dextran-SPION)		MRI quantification of infiltration of the pancreatic islet by monocytes/macrophages that had taken NPs in patients with T1D.	[25]
	Ultrasmall superparamagnetic iron oxide NPs (USPIOs)	Multiple sclerosis (MS)	After ingesting USPIOs, monocytes/macrophages can be visualized by MRI in the brains of rats and mice with EAE.	[26,27,28]
			MRI quantification of monocytes infiltration that had taken NPs in the CNS of patients with MS.	[[29], [30], [31]]
	USPIOs	Rheumatoid arthritis (RA)	These NPs detected joint inflammatory foci by targeting monocytes/macrophages that overexpressed the FR-β receptor in mice and rabbit models of RA.	[32]
	SPIONs coated with folic acid-conjugated dextran• •			[33]
**Therapy**	Lipid NPs loaded siRNA-CCR2	Type 1 diabetes (T1D)	Intravenous injection of siRNA-CCR2-NPs to mice decreased monocyte migration to foci of inflammation and prolonged normoglycemia in mice with streptozotocin-induced diabetes after pancreatic islet transplantation.	[34]
	High-density lipoprotein-mimicking peptide-phospholipid scaffold nanoparticles loaded with curcumin	Multiple sclerosis (MS)	After ingesting curcumin-loaded NPs, monocytes crossed the BBB in EAE mice, inhibited the proliferation of microglia, and restricted the infiltration of other effector immune cells. Mice treated with these NPs showed lower morbidity and mortality than those that received free curcumin.	[35]
	NPs of poly(dl-lactide-*co*-glycolide with high molecular weight•		Nanoparticles induced a significant reduction in the disease score and the number of myeloid cells and CD4^+^ T-cells in the CNS in mice with EAE.	[36]
	Poly(amidoamine) dendrimer nanoparticle covalently conjugated to polyvalent folic acid loaded with methotrexate	Rheumatoid arthritis (RA)	NPs reduced ankle diameter, paw weight, and total body weight in rats with collagen-induced arthritis compared to animals given free methotrexate.	[37]
	Folate-conjugated chitosan-glycol nanoparticles loaded with methotrexate		Folate-conjugated chitosan-glycol nanoparticles loaded with methotrexate reduced ankle diameter, paw thickness, and arthritis in adjuvant-induced arthritis rats compared with animals injected with free methotrexate or phosphate buffered saline.	[38]
	Mineralized nanoparticles loaded with methotrexate		Mineralized nanoparticles loaded with methotrexate ameliorated inflammatory arthritis in mice with collagen-induced arthritis.	[39]
	Chitosan NPs loaded with siRNA targeting the gene encoding TNF-α (siRNA-TNF-α)		NPs reduced TNF-α production by peritoneal macrophages and induced a reduction in local and systemic inflammation in mice with collagen-induced arthritis.	[40]
	PLGA NPs loaded with siRNA-TNF-α			[41]
	Micelle consisting of low-molecular-weight polyethylenimine–cholesterol–polyethylene glycol loaded with siRNA targeting NF-κB p65		NPs reduced cytokine production by macrophages *in vitro* and ameliorated inflammatory arthritis in mice collagen-induced arthritis model.	[42]

## 2 Use of nanoparticles in the diagnosis of autoimmune diseases

Early and accurate diagnosis of autoimmune diseases is necessary to treat patients properly. NPs have unique optical and physicochemical properties that allow them to be used in diagnostic procedures. For example, NPs can be utilized in molecular diagnostics to detect specific biomarkers of autoimmune diseases, such as specific DNA/RNA sequences and single nucleotide polymorphisms [18]. Carbon-based nanomaterials have been used as implantable nanosensors to track and detect blood glucose levels in patients with diabetes [19]. Additionally, NPs can act as contrast media in MRI.

### 2.1 *In vivo* imaging - animal models and patients with autoimmune diseases

*In vivo,* imaging has become an effective approach for early diagnosis, severity assessment, and therapeutic efficacy monitoring in various chronic diseases, including autoimmune disorders. *In vivo,* imaging makes it possible to localize and measure specific molecular targets and assess the physiology of a particular tissue or organ [43]. In many autoimmune disorders, early inflammation is usually asymptomatic; however, it involves activation of the endothelium that, in turn, increases vascular permeability, the expression of adhesion molecules, and the tissular infiltration by different cells of the immune system [44].

MRI is a valuable method for diagnosing autoimmune diseases because it detects inflammation-associated changes such as increased vascular permeability and cellular infiltration. This procedure frequently employs different contrast media administered intravenously and accumulating in inflamed organs. The contrast media primarily used in daily clinical practice are based on gadolinium and a chelating agent. Gadolinium is a heavy metal belonging to the lanthanide or rare earth family, and it is one of the most paramagnetic elements because it has many unpaired electrons. The chelating agent confers to the contrast media pharmacokinetic properties that facilitate their administration, metabolism, and elimination; and significantly decrease their toxicity, biological interactions, and deposition in tissues. Gadolinium-based contrast media transiently accumulate in inflamed tissues and enhance magnetic resonance imaging due to their ability to shorten the T1 relaxation time [45]. However, they have some limitations, such as low specificity, short half-life in circulation, and association with adverse effects such as nephrogenic systemic fibrosis [43]. Consequently, the development of new NP-based contrast media has been encouraged. Many studies have evaluated iron oxide NPs due to their high biocompatibility and superparamagnetic properties [46]. SPION are widely used as contrast media for MRI and are referred to as T2 or negative contrast agents. These NPs cause a magnetic field gradient that affects the surrounding protons of water molecules, thus altering the homogeneity of the magnetic field, which can be measured and observed by MRI. The Food and Drug Administration (FDA) approved the clinical use of several SPION preparations, including Ferumoxtran (dextran-SPION, 120–180 nm diameter), Ferucarbotran (carboxy dextran-SPION, 45–60 nm diameter), and Ferumoxtran-10 (dextran-SPION, 20–40 nm diameter), due to their low toxicity and biodegradability [47].

The use of different NPs preparations targeting monocytes/macrophages in the diagnosis of different autoimmune diseases MRI is described below.

#### 2.1.1 Type 1 diabetes (T1D)

Type 1 diabetes (T1D) is an autoimmune disease that results from the destruction of insulin-secreting β-cells in the pancreatic islets of Langerhans by autoreactive CD4^+^ T cells upon recognition of one or more β-cell peptides. Throughout the onset and progression of T1D, the pancreatic microvasculature changes, such as modification of the endothelium, transient vasoconstriction, vasodilatation, and increased blood flow and vascular permeability. These alterations favor the infiltration of the islets of Langerhans by lymphocytes, monocytes, and macrophages. The resulting insulitis promotes β-cell death and reduces insulin production [48]. An obstacle that hinders the diagnosis of T1D is the appearance of clinical manifestations when the vast majority of β cells have been destroyed, and the pancreas no longer produces enough insulin to control blood glucose levels. For this reason, efforts have been made to develop tools that make early diagnosis possible.

One of the most studied animal models of T1D is the non-obese diabetic (NOD) mouse that expresses the BDC2.5 T-cell receptor (TCR) transgene (rearranged TCR α (*Vα1*) and β (*Vβ4*) chain genes) [49]. These mice have autoreactive T-cells that recognize and respond uniformly to chromogranin A, a member of the grain family that plays an essential role in hormone secretion processes. Chromogranin A is involved in the generation of insulin-containing secretory granules in the β-cells of the pancreas [50]. Upon cleavage, chromogranin A generates several proteins, including WE-14, pancreastatin, and catestatin, which regulate carbohydrate metabolism [51]. Chromogranin A -derived self-peptides are presented by H2-II Ag7 molecules to self-reactive T-cells that invade the pancreatic islets and cause insulitis. The onset and progression of insulitis are more synchronous in these TCR BDC2.5 transgenic NOD mice than in NOD mice, making it easier to follow the different stages of the disease. During the first two weeks of life, the autoimmune process is absent. However, shortly thereafter, BDC2.5^+^ T-cells abruptly invade the pancreatic islets and produce rapidly progressive insulitis between the second and third weeks of life [48].

**Fig. 2:**
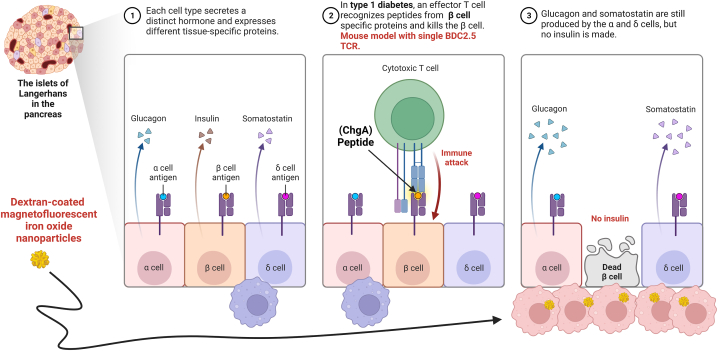
**Dextran-coated magnetofluorescent iron oxide nanoparticles are useful in the diagnosis of type 1 diabetes.** The development and progression of type 1 diabetes involve the destruction of insulin-secreting β-cells in the pancreatic islets of Langerhans. This destruction is caused by autoreactive CD4^+^ T cells that recognize one or more β-cell peptides, including Chromogranin A (ChgA). As a result, the pancreatic islets become infiltrated by lymphocytes, monocytes, and macrophages. This infiltration leads to insulitis, which further promotes β-cell death and reduces insulin production. In a mouse model of type 1 diabetes (NOD BDC2.5), the administration of dextran-coated magnetofluorescent iron oxide NPs intravenously resulted in their accumulation in the pancreas. These nanoparticles were captured by macrophages in the tissue and enabled the visualization of microvascular leakage, serving as an indicator of inflammation in the pancreata of the mouse models of type 1 diabetes. Created with BioRender.com.

MRI can detect changes in the pancreatic microvasculature noninvasively because the increased vascular permeability favors the accumulation of NPs that modify the tissue contrast. Denis et al. used dextran-coated magnetofluorescent iron oxide NPs with a long circulation time (>10 h) to detect early changes in the pancreatic microvasculature of 4-week-old NOD mice. Following intravenous administration, NPs accumulated in the pancreas after being captured by macrophages in the tissue. The accumulation of NPs was more significant in the pancreas of NOD BDC2.5 mice than in that of Eα16/NOD (MHC-II Eα: I-Eβ) mice without insulitis. A positive correlation was found between NPs accumulation and tissue injury caused by the inflammatory process [23] (Fig. 2). In 2005, a similar study reported that intravenous administration of monocrystalline superparamagnetic iron oxide NPs (22 nm diameter) to NOD BDC2.5 mice made it possible to detect inflammatory lesions *in vivo* by MRI. Furthermore, the study included a real-time evaluation of the therapeutic response in mice treated with a monoclonal antibody targeting the CD3ε subunit of the TCR. For this purpose, 4, 8, and 18 days after initiation of immunotherapy, mice were injected with NPs, and 24 h later, MRI was done. Monitoring of changes in pancreatic inflammation by MRI detected responses as early as day 8 of therapy; for example, the decrease in vascular permeability was more significant in mice that had received monoclonal mouse anti-CD3 than in mice treated with an isotype control. Moreover, mice treated with monoclonal anti-CD3 responded favorably, as evidenced by normalization of blood glucose levels within 2–4 weeks following treatment [24]. These studies demonstrate the utility of NPs to detect early changes in the permeability of the pancreatic microvasculature associated with the onset of T1D and to monitor treatment efficacy.

Some studies have demonstrated the use of NPs as contrast agents for diagnosing and MRI monitoring patients with autoimmune diseases, including T1D. Gaglia et al. conducted a study where they detected pancreatic islet inflammation in T1D patients using NPs-MRI. In this study, a cohort of 10 patients with recently diagnosed T1D (within six months) and 12 control subjects received an injection of Ferumoxtran-10 (dextran-SPION, 20–40 nm in diameter) before pancreatic analysis by MRI. The images obtained from T1D patients and controls showed significant differences; changes in the microvasculature and infiltration of the tissue by monocytes/macrophages that had taken up the NPs evidenced the presence of inflammatory lesions in the pancreas of patients with T1D [25].

#### 2.1.2 Multiple sclerosis

Multiple sclerosis (MS) is a chronic autoimmune inflammatory disease characterized by a loss of the blood-brain barrier integrity and the development of demyelinating lesions in the brain and spinal cord that are associated with neurological axonal injury. Patients with MS present with central nervous system (CNS) inflammation characterized by infiltration of peripheral immune cells, including monocytes, neutrophils, NK-, B-, and T-cells [52]. In experimental autoimmune encephalomyelitis (EAE), an animal model of MS, several phases of tissue inflammation are induced, followed by persistent myelin sheath damage in the CNS [26]. This animal model can be used to design or test new therapeutic strategies for MS. MRI with gadolinium chelates as contrast agents have been widely used as a diagnostic tool for MS. However, gadolinium-MRI is limited to visualization of the secondary effects of inflammation, *e.g*., gliosis and loss of blood-brain barrier integrity, as evidenced by diffusion of the gadolinium to the intercellular space.

Recently, magnetic NPs have been used as contrast agents to monitor *in vivo* the tissue distribution of phagocytes and detect for example their infiltration in the CNS. For this purpose, NPs are functionalized by conjugation with molecules that specifically bind to phagocyte membrane receptors. The NPs internalized confer sufficient magnetization to the cells to be detected by MRI or manipulated by an external magnetic field. Phagocytes internalize NPs by endocytosis or phagocytosis. However, uptake depends on the size, charge, and, mainly, surface functionalization of the NPs [53].

Inflammation in the CNS is characterized by widespread activation of mononuclear phagocytes, including both monocyte-derived macrophages and resident microglia cells. During EAE, the CNS is extensively infiltrated by inflammatory monocytes and macrophages. These cells produce cytokines such as interleukin (IL)-1β, IL-6, and IL-23, which promote the generation and maintenance of Th17 cells. Th17 cells are crucial in the development of CNS autoimmunity in the EAE model. Therefore, visualization of accumulated monocytes and macrophages in brain tissue is relevant. Several studies have shown that after ingesting dextran coated USPIO, monocytes/macrophages can be visualized by MRI in the brains of rats and mice with EAE [26,27,28]. A subsequent study in this animal model, compared the results of brain MRI scans done with different contrast media, gadolinium (T1-weighted), conventional (T2-weighted), and USPIO (T2-weighted). USPIO-MRI showed a higher sensitivity to detect brain lesions. It revealed the presence of tissue lesions characterized by a perivascular cellular infiltrate at sites where conventional or gadolinium MRI was negative. Moreover, histological analyses confirmed the presence of macrophages at sites of tissue injury detected by MRI [27]. Rausch et al. obtained similar results in a study with the EAE model in Lewis rats immunized with guinea pig myelin. The authors demonstrated that systemically administered USPIO were internalized by monocytes located in the CNS [26].

Recently, the characterization of tissue lesions using NPs-based contrast media has been implemented in patients with MS. The accumulation of USPIO in phagocytic cells allows for the *in vivo* detection of infiltrating macrophages in the brain parenchyma. Dousset et al. evaluated the brain lesions in 10 patients with relapsing-remitting MS by MRI. Patients received intravenous injections of USPIO and gadolinium to detect different aspects of the CNS inflammatory process. The uptake of USPIO by circulating monocytes made it possible to visualize their activity *in vivo* and their presence in the CNS cell infiltrate. On the other hand, the gadolinium revealed the increased permeability of the blood-brain barrier [29]. Furthermore, some experimental evidence suggests that, compared to the gadolinium, USPIO confers greater sensitivity to MRI in detecting the number of CNS lesions [30,31]. In 2008 Vellinga et al. studied 19 patients with relapsing-remitting MS and observed that in 14 individuals with active disease, MRI showed 188 USPIO-positive lesions, of which 144 were gadolinium negative [30]. Additionally, Tourdias et al. demonstrated that the combination of both contrast agents (USPIO and gadolinium) helped to detect lesions that were missed when gadolinium was used alone (increased detection rate of 51%) [31]. The additional information provided by NPs to gadolinium-enhanced MRI may be related to monocyte infiltration of the CNS. Therefore, USPIO-enhanced MRI could provide a better understanding of the complexity of the inflammatory process in multiple sclerosis.

#### 2.1.3 Rheumatoid arthritis

Rheumatoid arthritis (RA) is a chronic inflammatory systemic disease characterized by massive destruction of bone and cartilage and inflammation of joint synovial tissue [54]. Macrophage infiltration of the synovium is one of the most important features of RA. Numerous investigations have shown that the frequency and the absolute number of macrophages are significantly increased in the affected synovial tissues of patients with RA [55]. The infiltrating macrophages secrete high levels of pro-inflammatory cytokines such as tumor necrosis factor-alpha (TNF-α) and IL-1β that participate in joint destruction by promoting the activation of synovial fibroblasts and the production of matrix metalloproteinases [32]. The destructive nature of the disease, as evidenced by irreversible cartilage and bone damage, highlights the need for an early diagnosis. X-ray imaging is the primary tool for diagnosing RA and monitoring the progression of joint destruction. However, it can only detect late signs of the disease, making it difficult to prevent irreversible bone damage in a high proportion of patients. On the other hand, MRI makes it possible to detect the early stages of RA. In this case, NPs can passively accumulate in the chronically inflamed tissue due to the enhanced permeability and retention effect. Furthermore, the diagnostic efficacy of MRI for RA can be improved by functionalizing NPs with ligands of receptors overexpressed on the membrane of the inflammatory macrophages that infiltrate the synovial tissue. In addition, *in vivo* detection of activated macrophages in the affected joints by MRI is a valuable tool for monitoring the response to therapeutic agents.

NPs can be functionalized with folic acid to bind to specific cell receptors. Folate receptors comprise a family of cell surface-anchored molecules with a high affinity for folic acid. There are three folate receptors isoforms in humans, -α, -β, and -γ. The folate receptor-β is overexpressed on activated macrophages that are involved in RA, Crohn's disease, SLE, and psoriasis. Dai et al. used adjuvant-induced arthritis model in Lewis rats (subcutaneous paw injection of 0.1 mL of complete Freund's adjuvant) to evaluate the detection of inflamed tissues by NP-MRI. For this purpose, they designed glucose-containing SPIONs coated with folic acid-conjugated dextran. These NPs detected joint inflammatory foci more effectively by targeting monocytes/macrophages that overexpressed the folate receptor-β. Moreover, *in vitro,* assays with murine RAW 264.7 macrophages, previously activated with TNF-α and IFN-γ, revealed that glucose-containing SPION coated with folic acid-conjugated dextran were more efficiently ingested than non-conjugated NPs [33]. Lutz et al. also demonstrated the efficacy of USPIO-MRI to detect activated macrophages in a rabbit model of RA induced by intraarticular (knee) injection of methylated bovine serum albumin. Rabbits received an intravenous injection of USPIO NPs, and 24 h later, knee images were obtained by MRI. They evidenced the uptake of the NPs by activated macrophages present in the swollen joints [32].

The findings in the animal models and patients described above have opened the possibility of using NPs as tools to diagnose and assess the severity of inflammatory lesions in autoimmune diseases.

## 3 Use of nanoparticles in the treatment of autoimmune diseases

The treatment of autoimmune diseases is focused on controlling the immune response, either with immunosuppressive drugs that induce antigen tolerance or by enhancing the activation of the immune response. The potential of NPs has been evaluated in both scenarios.

### 3.1 Nanoparticles as drug carriers-animal models

The use of biodegradable NPs to transport different drugs, peptides, and oligonucleotides has been extensively evaluated *in vitro* and *in vivo.* These NPs have several advantages, such as biocompatibility, increased cargo uptake rate, and sustained cargo release. They also make it possible to overcome biological barriers, thus favoring cargo delivery to the target cells, tissues, or organs at the appropriate concentrations.

#### 3.1.1 Multiple sclerosis

The FDA has approved several drugs for treating patients with MS, such as glatiramer acetate, dimethyl fumarate, natalizumab (anti-VLA-4), and alemtuzumab (anti-CD52 antibody). However, all of them are associated with different side effects. One of the main obstacles to treating patients with diseases of the CNS is the inability of most therapeutic agents to cross the blood-brain barrier. Therefore, NP-based strategies are under development to facilitate the passage of molecules across it [35,56].

**Fig. 3:**
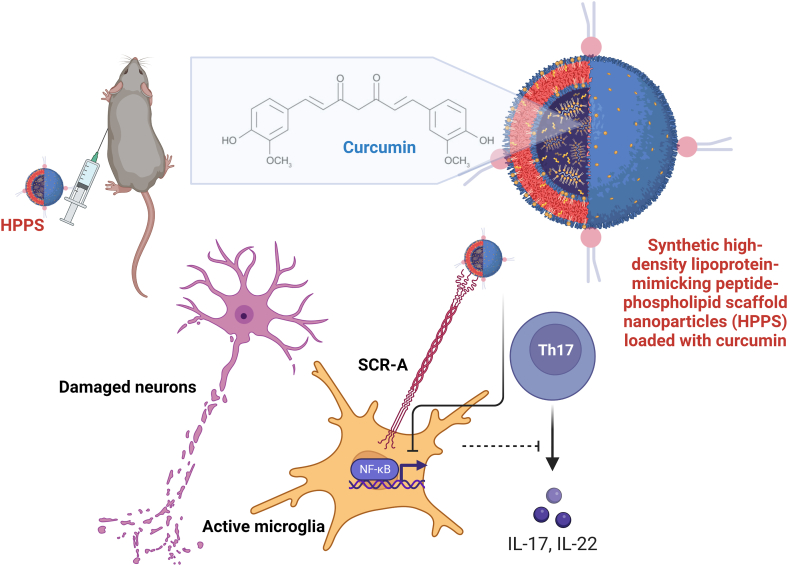
**Synthetic high-density lipoprotein-mimicking peptide-phospholipid scaffold NPs loaded with curcumin (in the figure designed as** cur-HPPS**) delays the progression of experimental autoimmune encephalomyelitis.** Monocytes ingested these NPs through the scavenger receptor class B type I receptor in experimental autoimmune encephalomyelitis mice. Mice treated with these NPs showed lower morbidity and mortality than those that received free curcumin. These NPs inhibited the infiltration of immune cells and protected the myelin sheath from damage by activated immune cells. These results were attributed to the ability of the NPs to modulate inflammatory monocytes by inhibiting the nuclear factor kappa B (NF-κB) pathway. Created with BioRender.com.

As described above, monocytes play an essential role in the onset and evolution of MS because they cross the blood-brain barrier, promote neuronal damage, and recruit other immune system cells into the CNS. Lu et al. injected EAE mice with synthetic high-density lipoprotein-mimicking peptide-phospholipid scaffold NPs loaded with curcumin to evaluate their immunomodulatory activity on inflammatory monocytes. They found that monocytes ingested these NPs through the scavenger receptor class B type I in both *in vivo* and *in vitro* experiments. Mice treated with curcumin-loaded NPs showed lower morbidity and mortality than those that received free curcumin. These results were attributed to the ability of these nanoparticles to modulate inflammatory monocytes by inhibiting the nuclear factor kappa B (NF-κB) pathway and decreasing the expression of adhesion and migration-related molecules, such as ICAM-1 and Macrophage-1 antigen (Mac-1) [35] (Fig. 3). Also, in the EAE mouse model, Saito et al. evaluated drug-free NPs synthesized from 50:50 PLGA with low or high molecular weight or PLA with low molecular weight. Specifically, they assessed the ability of these NPs to associate with circulating monocytes and neutrophils, thereby inhibiting their migration to the CNS and their impact on the course of the disease. *In vitro* and *in vivo* experiments showed that neutrophils and monocytes effectively took up the NPs. Furthermore, intravenous treatment with PLGA with high molecular weight NPs induced a significant reduction in the disease score and the number of myeloid cells and CD4^+^ T-cells in the CNS [36]. These findings demonstrated that NPs are a promising tool to target cells of the innate immune system, such as inflammatory monocytes, and reduce their traffic to tissues injured or inflamed.

#### 3.1.2 Rheumatoid arthritis

**Fig. 4:**
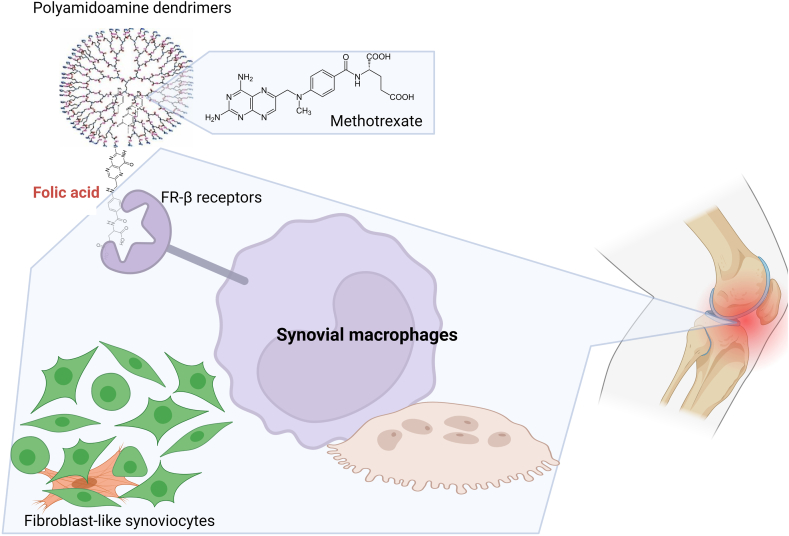
**Polyamidoamine dendrimers conjugated with folic acid loaded with methotrexate are efficient in the treatment of inflammatory arthritis.** Methotrexate loaded NPs were recognized by folate receptor-β, whose expression is selectively elevated in synovial macrophages of patients with RA. Intravenous administration of these NPs to rats with collagen-induced arthritis) reduced ankle diameter, paw weight, and total body weight compared to animals given free methotrexate. Created with BioRender.com.

Regarding RA, therapeutic agents are intended to reduce joint inflammation and damage. However, not all patients respond effectively to these drugs; some have adverse side effects, such as hair loss, headache, and lung, liver, and kidney toxicity. Therefore, several studies have evaluated the therapeutic potential of NPs to target the drug specifically to the cells involved in the inflammatory process [37,38]. Since macrophages infiltrate the inflamed synovial membrane and cartilage and play a critical role in RA pathogenesis, the design of NPs that specifically target these cells has been widely considered. Thomas et al. demonstrated *in vitro* that RAW 264.7 macrophages and macrophages isolated from the peritoneal cavity of mice efficiently uptake methotrexate encapsulated in poly amidoamine dendrimers covalently conjugated with folic acid. Methotrexate-loaded NPs were recognized by folate receptor-β, whose expression is selectively elevated in synovial macrophages of patients with RA. They also demonstrated the therapeutic potential of methotrexate-NPs when administered intravenously to rats with collagen-induced arthritis. A favorable response was evidenced by the significant reduction in ankle diameter, paw weight, and total body weight in rats with collagen-induced arthritis compared to animals given free methotrexate [37] (Fig. 4). In 2020, another study evaluated the characteristics and therapeutic potential of folate-conjugated chitosan-glycol NPs loaded with methotrexate in the Wistar rat model with adjuvant-induced arthritis. Such NPs were stable in serum and did not induce hemolysis of rat red blood cells. Lipopolysaccharide (LPS)-activated murine RAW 264 macrophages took up these NPs via folate receptor-β to a greater extent than non-activated macrophages; human embryonic kidney (HEK) cells and mouse embryonic fibroblasts (NIH-3T3) did not take up the NPs. Interestingly folate-conjugated chitosan-glycol NPs loaded with methotrexate ingested by macrophages induced reactive oxygen species (ROS) production and reduction of antioxidant enzymes that led to cell apoptosis [38]. Additional *in vivo* experiments showed that, after intravenous administration of ^99m^Tc radiolabeled folate-conjugated chitosan-glycol NPs loaded with methotrexate to adjuvant-induced arthritis rats, the NPs accumulated in high concentrations in arthritic joints. Furthermore, NPs induced a reduction in ankle diameter, paw thickness, and arthritis score that were superior to those observed in rats injected with free methotrexate or phosphate-buffered saline [38]. These results suggest that specific uptake of methotrexate-loaded NPs by inflammatory macrophages through folate receptor-β receptors can suppress the inflammatory changes associated with arthritis.

Another study described the therapeutic properties of mineralized NPs composed of PEGylated hyaluronic acid as a hydrophilic layer, 5β-cholanic acid as a hydrophobic core, and calcium phosphate as a pH-sensitive mineral. These particles can release cargo across neutral to acidic conditions as those of inflamed joints. *In vitro,* assays showed that macrophages internalized these particles through endocytosis mediated by different molecules, mainly CD44, stabilizing-2, and the receptor for hyaluronan-mediated motility. In addition, these NPs were loaded with methotrexate to verify drug release at different pH conditions (from 7.4 to 5.0); the drug release was higher as the pH decreased. Moreover, symptom relief was observed when mineralized NPs were conjugated with methotrexate and injected into mice with collagen-induced arthritis [39]. Thus, the transport of methotrexate on NP-based platforms targeting sites of inflammation could reduce drug-associated side effects.

## 4 Nanoparticles as carriers for nucleic acids

Gene silencing with small interfering ribonucleic acid (siRNA) has been explored as a therapeutic strategy for the treatment of different diseases [57]. siRNAs can disrupt the translation process, silence genes, and even inhibit corresponding proteins' expression via targeting a specific messenger RNA (mRNA) [58]. However, the instability of siRNA under physiological conditions limits its therapeutic use. NPs have become an alternative for docking and transporting siRNA molecules to increase their stability and prevent their degradation by circulating nucleases. Previous studies have demonstrated that different types of NPs, including lipidic, polymeric, and inorganic NPs could be a fast and effective way to specifically target cell types with potential therapies such as siRNA in autoimmune conditions and other inflammatory diseases [58]. Howard et al. reported that intraperitoneal administration of chitosan NPs loaded with siRNA targeting the gene encoding TNF-α (siRNA-TNF-α) to mice with collagen-induced arthritis reduced TNF-α production by peritoneal macrophages and induced a reduction in local and systemic inflammation [40]. Similarly, a study published in 2012 demonstrated that PLGA NPs loaded with siRNA-TNF-α inhibited TNF-α expression in RAW 264.7 macrophages in a dose-dependent manner. Furthermore, administration of these NPs to DBA/1 J mice with collagen antibody-induced arthritis induced a reduction in paw inflammation and joint effusion [41]. In another related research, Chen et al. demonstrated that the encapsulation of siRNA targeting NF-κB p65 in cationic delivery micelle consisting of low-molecular-weight polyethyleneimine–cholesterol–polyethylene glycol, protected and delivered it to macrophages with high efficiency. The p65 siRNA/NPs complex inhibited macrophage-based cytokine release *in vitro* and efficiently suppressed arthritis development in a mouse collagen-induced arthritis model [42], thus demonstrating the therapeutic potential of siRNA-loaded NPs.

Since inflammatory monocytes depend on the chemokine receptor CCR2 to migrate to foci of inflammation, Leuschner et al. designed 70–80 nm lipid NPs containing siRNA-CCR2 and studied their effect after intravenous administration in mice with different inflammatory conditions (atherosclerosis, myocardial infarction, pancreatic islet transplantation in diabetes, and cancer induced by EL4 lymphoma cell implantation). The CCR2 knockdown was achieved in Ly-6C^high^ monocytes isolated from the spleen and was confirmed at the mRNA and protein levels. In addition, intravenous injection of siRNA-CCR2-NPs to mice decreased monocyte migration to foci of inflammation and thus produced favorable effects in the different models studied: (i) attenuated the number of atherosclerotic plaques, thus reducing the myocardial infarct size after coronary artery occlusion; (ii) prolonged normoglycemia in diabetic mice after pancreatic islet transplantation; and (iii) reduced tumor volume and the number of tumor-associated macrophages in the EL4 lymphoma model; interestingly, TAMs inversely correlates with survival in patients with lymphoma [34].

## 5 Nanoparticles as drug carriers - human monocytes

Although the FDA has accepted the clinical use of some NPs, such as SPION, more experimental evidence is still needed for other NPs to be used as therapeutic tools to treat patients with different diseases. As the design of new NPs progresses, it becomes necessary to evaluate their effect on human cells. Several studies have shown how NPs interact with and affect different human cells, including some of the immune systems, such as lymphocytes, monocytes, macrophages, and dendritic cells. The preponderant role of monocytes and macrophages in the onset and progression of autoimmune diseases has made them attractive targets for diagnosing and treating these conditions. On the other hand, the interaction of NPs with circulating proteins, such as opsonins and other blood components, favors their recognition and internalization by monocytes and macrophages [59]. These interactions also play a fundamental role in the biodistribution of NPs since, after being ingested by monocytes that migrate to infected or inflamed tissues, the NPs can accumulate to a greater extent in these compromised areas [60].

To develop contrast media for selective and differential tracking of human monocytes *in vivo*, Giraldo-Villegas et al. evaluated the features of polyacrylate-coated iron oxide NPs. They found that these NPs were captured by monocytes through scavenger receptors and did not affect their viability, differentiation to macrophages, or ability to phagocytose latex spheres [61]. Since monocytes efficiently capture polyacrylate-coated iron oxide NPs without adverse effects on their phenotype and function, they could be a valuable tool for characterizing early tissue injury and understanding their role in different immunopathologies, including autoimmune diseases.

Chitosan NPs have also been explored for targeting monocyte/macrophages. Chitosan is a natural and biocompatible polymer that has been extensively studied in various biomedical applications, such as drug delivery and tissue engineering strategies. As chitosan could interfere with the phenotype and function of some cells, it is critical to understand its effect on macrophage characteristics. In 2012 Oliveira et al. evaluated for 10 days the polarization of human monocyte-derived macrophages on an ultrathin chitosan film and observed a reduced expression of the surface molecules CD86 and HLA-DR, reduced production of proinflammatory cytokines such as TNF-α, and increased production of the anti-inflammatory cytokines IL-10 and transforming growth factor-β1 (TGF-β). These results suggested that chitosan induces the polarization of macrophages towards the anti-inflammatory-M2 phenotype [62]. Subsequently, in 2015, the same group designed chitosan and γ-glutamic acid NPs loaded with diclofenac and evaluated their effect on human macrophages. These NPs were efficiently taken up by macrophages and inhibited prostaglandin E2 synthesis [63], *i.e*., they showed anti-inflammatory activity.

Another study by Rafique et al. described the design of polyethylene glycol-coated lipid NPs containing calcitriol and functionalized with anti-CD163 antibodies and evaluated their effect on human monocyte-derived macrophages. The particles were endocytosed by macrophages through the CD163 receptor and induced the reduction of NF-κB, TNF-α, monocyte chemoattractant protein-1 (MCP-1), and IL-6 mRNA levels; a lower secretion of TNF-α and IL-6; and the increase of IL-10 mRNA levels [64]. These data demonstrated that drug-loaded NPs could contribute to modulating the inflammatory function of monocytes and thus ameliorate local inflammatory reactions typical of autoimmune diseases.

### 5.1 Nanoparticles and monocyte subpopulations

At least three monocyte populations have been described in humans based on surface expression of CD14 (LPS co-receptor along with MD-2 and TLR4, which mediates LPS signaling) and CD16 (FcγIIIa receptor) as follows: classical (CD14^++^/CD16^-^), intermediate (CD14^+^/CD16^+^) and non-classical (CD14^+^, CD16^++^) monocytes. Several authors have described striking alterations in the proportion and the absolute number of circulating monocyte subpopulations in a wide range of patients with different diseases, including autoimmune diseases such as SLE [65,66] and RA [67]. Therefore, several studies have sought to elucidate the ability of different monocyte subpopulations to internalize NPs and their consequent effects. For instance, Settles et al. showed by flow cytometry that classical monocytes, isolated from human peripheral blood, took up superparamagnetic fluorescent iron oxide NPs more efficiently than non-classical monocytes. Furthermore, fluorescence microscopy assays showed that the uptake of NPs was clathrin-dependent, as evidenced by the colocalization of fluorescent signals from clathrin vesicles and particles. Internalized NPs altered the monocyte phenotype: classical monocytes showed increased expression of CCR2 and CD120a; non-classical monocytes exhibited increased expression of CD206 and decreased expression of the fractalkine receptor CX3CR1, and both cell subsets showed a higher expression of HLA-DR [68].

Wildgruber et al. compared the uptake of dextran-coated superparamagnetic iron oxide NPs coupled to macrophage colony-stimulating factor receptor (MCSFR) antibodies by classic and non-classic monocytes. They found that, although both cell subpopulations expressed similar levels of MCSFR, the uptake of NPs was better in classical monocytes and did not affect their viability [69]. This differential uptake of NPs by monocyte subpopulations could be used for *in vivo* detection and characterization of these cell subsets in different diseases.

## 6 Limitations and future perspectives of using nanoparticles to target monocytes and macrophages

Despite the advancements in nanomedicine and the promising results achieved with different formulations of NPs in diagnosing and treating various diseases, including autoimmune conditions, it is important to note that many of these formulations are still in the laboratory phase. Only a limited number of NP-based formulations have received approval for clinical use [70]; this is because there are still obstacles and challenges to overcome in this field: The careful design, rigorous characterization, and reproducible manufacturing process are crucial to achieving a consistent and reliable drug formulation using NPs. NPs are complex three-dimensional structures, where each component plays a specific role. Any alteration in the physicochemical properties such as charge, hydrophobicity, or size can have a profound impact on the specificity, drug release kinetics, biocompatibility, toxicity, and *in vivo* behavior of NPs [71]. Another limitation in the use of NPs is their rapid clearance from the bloodstream and accumulation in organs such as the liver and spleen. To overcome this challenge, the surface modification of NPs using polyethylene glycol has been extensively employed. Polyethylene glycol, due to its hydrophilicity, can inhibit the uptake of NPs by the reticuloendothelial system and reduce non-specific interactions with blood components [17]. However, the use of polyethylene glycol can trigger an immune response [72], leading to the production of anti-polyethylene glycol antibodies, which has been associated with decreased efficacy of PEGylated systems [73].

Recently, the coating of NPs with cell membranes has emerged as a novel therapeutic tactic to overcome this obstacle. These systems contain cell membrane molecules and maintain the targeting mechanisms of the progenitor cell, they can escape the immune system and remain in circulation for long periods [74]. Some studies have ventured into the use of this strategy for the design of NPs coated with membranes of various types of cells, including red blood cells, stem cells, cancer cells [74], monocytes [75], and macrophages [[76], [77], [78]], the latter migrate to tumors and inflamed tissues through surface receptors such as integrins, MAC-1 and CSF1R [76], exercising their function there.

Monocytes and macrophages play a significant role in autoimmune diseases, making them attractive targets for diagnosis and treatment. Although various NPs formulations with different ligands have been developed to enhance their specificity for these cells, there are still challenges that impede their clinical use. The difficulty lies in achieving specific labeling of monocytes/macrophages *in vivo* due to the shared markers and receptors with other immune cells. To address this, nanoparticle systems can be functionalized with a range of ligands to increase specificity. Moreover, considering that different populations of monocytes (classical, non-classical, and intermediate) and macrophages (M1 and M2) could play a differential role in the immunopathology of autoimmune diseases, the design of NPs targeting specific subpopulations could be highly valuable. However, these subpopulations share several receptors, which poses a challenge to achieving selectivity with NPs. Some studies point to the use of NPs as reprogrammers of the M1/M2 phenotype of macrophages, which contain mRNAs that code for transcription factors [79] and cytokines/chemokines [80], contributing to cell polarization.

Although there are challenges to overcome, the improvement in NPs synthesis and characterization techniques; as well as the understanding of their interaction with monocytes/macrophages, open new possibilities in the design of more specific and effective NPs for the diagnosis and treatment of autoimmune diseases.

## 7 Conclusion

Monocytes are a population of bone marrow-derived leukocytes that in response to chemotactic signals migrate to sites of inflammation, where they can differentiate into macrophages and dendritic cells. All these cells accomplish several essential immunological functions, including phagocytosis, antigenic presentation, production of soluble mediators, initiation, and resolution of inflammation, as well as recruitment of other cells of the immune system. Monocytes/macrophages play an essential role in the onset and progression of different autoimmune diseases, including SLE, RA, and MS. Moreover, these cells can efficiently internalize different types of NPs, cross nearly impermeable biological barriers, and migrate early to foci of inflammation. Due to these characteristics, several research groups have proposed that monocytes/macrophages be used as targets of superparamagnetic NPs for *in vivo* monitoring by MRI. Additionally, as NPs can be loaded with some drugs, peptides, and nucleic acids, they can be used not only to visualize the location of monocytes/macrophages *in vivo* but also to precisely modulate their inflammatory function. The experimental evidence described in this review, and summarized at the graphical abstract, supports the potential use of NPs in the early diagnosis and effective treatment of autoimmune diseases.

## Author contribution statement

All authors listed have significantly contributed to the development and the writing of this article.

## Funding statement

Ministry of Science, Technology and Innovation of Colombia (Project ID: 111584467267).

## Data availability statement

No data was used for the research described in the article.

## Declaration of competing interest

The authors declare that they have no known competing financial interests or personal relationships that could have appeared to influence the work reported in this paper.
